# PS-33 - COMMUNITY ACUTE RESPIRATORY INFECTION (CARI) SENTINEL SURVEILLANCE OF NON-COVID-19 SEASONAL CORONAVIRUSES IN SCOTLAND OVER A 4-YEAR PERIOD

**DOI:** 10.1093/pubmed/fdag046.070

**Published:** 2026-07-09

**Authors:** Ms Catríona Oliver, Dr Helen Gadegaard, Mr Mark Hamilton, Dr Josie Evans

**Affiliations:** Public Health Scotland; Public Health Scotland; Public Health Scotland; Public Health Scotland

## Abstract

**Background:**

Non-COVID-19 seasonal human coronaviruses (HCoV) cause ~10-30% of common colds, particularly during colder months. A complex interplay of factors (environmental, behavioural, immune-related) drive HCoV circulation, and the COVID-19 pandemic disrupted well-established patterns, with low levels of HCoV in 2020/21 and an early peak in 2021/22. Ongoing HCoV surveillance is important to i)monitor seasonal patterns contributing to health system pressures; ii)to follow trends in ARI cases with unknown aetiology for future pandemic monitoring.

**Objective:**

To describe post-pandemic HCoV circulation in Scotland.

**Methods:**

Since October 2022, the CARI sentinel surveillance system in >100 GP practices in Scotland has monitored HCoV infection, and 3 common endemic types. Selected symptomatic ARI cases (3 Oct 2022-8 March 2026) were recruited, tested for 10 respiratory pathogens, symptoms data collected and results linked to clinical datasets.

**Results:**

4.9% (4,380/90,467) of CARI-tested patients tested positive for HCoV; 753, 1,072 and 2,450 for alpha-types HCoV229e and HCoVNL63, and beta-type HCoVOC43 respectively (105 untyped). In 2022/23, peak test positivity for HCoV was 14.0% in ISOweek 51, but, more typically, peaks were higher and occurred in ISOweeks 6,8,10 respectively in the following three seasons. HCoVOC43 is annual and dominated every season. The biannual, alternate patterns of HCoV229e and HCoVNL63 are re-establishing. There were slightly different proportions of HCoV229e (low in 0–4s) and HCoVNL63 (high in 5–14s), otherwise distributions of type by age were similar. Co-infection with another pathogen was detected for 28.0% (1,226/4,380) of HCoV detections. There were 60 hospital admissions within 14 days of HCoV infection, 18 (30.0%) with co-infection detected. There were 6 deaths within 28 days (in 0–4 and 65+ age-groups), two with co-infection with influenza A.

**Conclusion:**

Typical HCoV circulation patterns are re-emerging. Co-infection with HCoV may increase risk of adverse outcomes in vulnerable patients exposed to other pathogens, contributing to winter pressures.

